# Retinal changes in children with Familial Mediterranean Fever: the effect of chronic subclinical inflammation

**DOI:** 10.1038/s41390-025-04195-7

**Published:** 2025-06-06

**Authors:** Nadide M. Sav, Kuddusi Teberik

**Affiliations:** 1 Department of Pediatric Nephrology, Duzce University, Duzce, Turkey; 2 Department of Ophthalmology, Duzce University, Duzce, Turkey

## Abstract

**Background:**

In Familial Mediterranean Fever (FMF), acute-phase reactants rise during attacks, indicating active inflammation. However, subclinical inflammation—present even during remission—may contribute to organ damage, including ocular involvement. This study aimed to investigate the effects of subclinical chronic inflammation on ocular structures in children with FMF.

**Methods:**

The study involved 51 pediatric FMF patients in remission for at least three months and healthy controls. Spectral domain optical coherence tomography (SD-OCT) was used to measure intraocular pressure, axial length, peripapillary retinal nerve fiber layer (RNFL) thickness, central macular thickness, and subfoveal choroidal thickness.

**Results:**

Acute-phase reactant levels were significantly elevated in the FMF group (*p* < 0.001). Inferotemporal RNFL thickness was notably reduced (*p* = 0.008), along with central macular, subfoveal, nasal, and temporal choroidal thicknesses. A mild positive correlation was observed between proteinuria and axial length (*r* = 0.282, *p* = 0.045).

**Conclusion:**

Subclinical inflammation in FMF may lead to early structural changes in the eye, potentially progressing over time. These findings highlight the importance of long-term ophthalmologic monitoring in pediatric FMF patients to better understand the cumulative impact of persistent inflammation.

**Impact:**

- This study assessed peripapillary RNFL thickness and choroidal vascular structure in children with FMF using SD-OCT to evaluate ocular effects of subclinical inflammation.
- It was found that chronic inflammation may lead to thinning of the retina and choroid even in children with relatively short disease duration.
- Peripapillary RNFL thickness was reduced in the temporal inferior quadrant and correlated with serum amyloid levels; additionally, choroidal thickness was significantly lower in the patient group, suggesting early ocular involvement and its potential as a marker of subclinical systemic inflammation.

## Introduction

Familial Mediterranean fever (FMF) is an inflammatory disease characterized by recurrent, self-limiting attacks of fever and serositis.^1^ While inflammation during attacks is the primary cause of amyloidosis and irreversible organ damage, subclinical inflammation persisting during attack-free periods may also contribute to tissue and organ injury.

The gene responsible for FMF, named MEFV by the International FMF Consortium, encodes a protein known as pyrin or marenostrin.^2,3^ Pyrin plays a regulatory role in inflammation, particularly through its autoregulatory effects on leukocytes.^4^ In FMF, missense mutations in the MEFV gene alter the structure and function of pyrin, impairing its role in the regulation of innate immunity.^5^ These mutations lead to activation of the caspase-1 enzyme, resulting in increased release of interleukin (IL)-1β.^6^ IL-1β subsequently promotes the production and activation of tumor necrosis factor-alpha (TNF-α). These proinflammatory cytokines contribute to the chronic inflammatory state characteristic of FMF.^7^

This persistent inflammatory response may also impact ocular tissues, particularly those with rich vascular structures. Disease-related changes occur in retinal and choroidal vascular structures because FMF triggers vasculopathy and inflammation.^8^ Notably, the choroid is considered more susceptible to systemic inflammatory and vascular conditions compared to other ocular structures.^9,10^ In cases where excessive inflammation is triggered, such as COVID-19, changes can also be observed in retinal nerve fibers and ganglion cells with complex vascular structures.^11^ In addition, IL-1β, the release of which increases during the disease, can activate TNF-α, and together they can affect retinal structures.^6,7,9^ The effects of these proinflammatory cytokines have been extensively studied. An experimental study by Campagno et al. showed that IL-1β derived from microglial cells can increase chemokine levels in retinal pigment epithelial cells and Müller cells and contribute to retinal degeneration. The researchers stated that a better understanding of these pathways may provide new therapeutic targets for retinal pathologies, especially considering the central role of IL-1β in initiating inflammatory cascades in chronic autoinflammatory diseases.^11^ The proinflammatory cytokine, IL-1β, has been demonstrated to play a role in maintaining immune responses and contributing to disease severity in a variety of central nervous system diseases, including multiple sclerosis, neurodegenerative diseases, traumatic brain injury, and diabetic retinopathy. However, pharmacological blockade of IL-1 signaling has been demonstrated to be advantageous in certain autoimmune and autoinflammatory diseases.^12^

Due to the risk of serious complications associated with invasive eye examinations, non-invasive imaging techniques have gained importance. Spectral-domain optical coherence tomography (SD-OCT) is a non-contact, non-invasive, transpupillary imaging modality used to assess retinal architecture. Since Spaide et al. described enhanced depth imaging (EDI)-OCT, choroidal thickness (CT) has been extensively evaluated in various chorioretinal disorders using this technology.^13^

Considering the hypothesis that FMF-related inflammation may persist during asymptomatic periods and continue to affect ocular tissues, this study aimed to assess peripapillary retinal nerve fiber layer (RNFL) thickness and choroidal vascular structures in pediatric FMF patients using SD-OCT, with a focus on identifying potential ocular manifestations of subclinical inflammation.

## Materials and methods

This study was conducted with 51 pediatric patients diagnosed with FMF and 51 healthy controls. Written informed consent was obtained from all participants and their families. Ethical approval was granted by the local ethics committee (Ethics No: 2020/230). FMF was diagnosed according to Tel-Hashomer diagnostic criteria.^14^ In addition, disease severity was measured using the International Severity Scoring System for Familial Mediterranean Fever (ISSF).^15^

All examinations and imaging procedures were performed on patients who had been in remission for at least three months. The average remission period of the patients was also assessed. All examinations and imaging procedures were conducted during a remission period of at least three months, and the average remission duration was recorded. All patients were on regular colchicine therapy. Physical examinations were unremarkable in all participants.

Exclusion criteria included: age outside the 6–18-year range, history of ocular surgery or trauma, congenital ocular anomalies, amblyopia, refractive errors exceeding ±1.0 diopter, intraocular pressure ≥21 mmHg, glaucomatous optic disc changes, history of contact lens use, inflammatory ocular diseases such as strabismus, uveitis, scleritis, corneal scars or ectasia, use of topical ocular medications, optic nerve disorders, amyloidosis, autoimmune or vasculitic diseases, and systemic comorbidities such as chronic kidney disease, diabetes mellitus, or hypertension.

All ocular assessments were performed on the right eye of each participant. Comprehensive ophthalmologic evaluation included best-corrected visual acuity, refraction measurement via an auto kerato-refractometer (Topcon KR-8100, Topcon Corporation, Tokyo, Japan), intraocular pressure measurement using a non-contact tonometer (Canon TX-20P), and axial length assessment with the IOL-Master 700 (Carl Zeiss Meditec, Jena, Germany). Dilated fundus examination was performed with a 90-diopter lens.

Macular, RNFL, and choroidal thicknesses (CT) were evaluated using Spectral Domain Optical Coherence Tomography (SD-OCT, Heidelberg Engineering, Heidelberg, Germany). All measurements were performed using the device’s built-in software, with manual adjustment of anatomical landmarks such as the sclerochoroidal interface to enhance accuracy. Optic disc boundaries were delineated manually by marking seven points along the inner margin of the scleral ring. The RNFL thickness was measured globally (G) and in six sectors: temporal (T), superotemporal (ST), superonasal (SN), nasal (N), inferotemporal (IT), and inferonasal (IN) quadrants.

Choroidal thickness was defined as the perpendicular distance between the outer border of the retinal pigment epithelium (automatically detected) and the manually marked sclerochoroidal interface. Retinal thickness (RT) and CT were measured at seven points: one subfoveal, three nasal, and three temporal locations, at 500-μm intervals extending up to 1500 μm.^16^ RT and RNFL thicknesses were assessed automatically, whereas CT measurements were performed manually. All choroidal assessments were conducted in enhanced depth imaging (EDI) mode, using the High-Speed (HS) scanning protocol. Only horizontal B-scans were analyzed; volume scans were excluded. The methodology was based on previously published studies.^17,18^

To minimize diurnal variation, all OCT scans were performed between 09:00 and 11:00 AM by a single experienced ophthalmologist. Each measurement was performed twice during the same session, and the average value was used for statistical analysis to ensure reproducibility.

Blood and urine samples were stored under appropriate conditions, and all laboratory analyses were conducted concurrently.

### Statistical analysis

The distribution of the data was analyzed by the Kolmogorov–Smirnov test and the homogeneity of variance by Levene’s test. The groups were compared using the Independent Samples *t*-test, Welch test, One-Way ANOVA or Mann–Whitney U and Kruskal–Wallis tests, depending on the type of data distribution and the number of groups compared. Categorical variables were analyzed by Pearson’s Chi-Square, Fisher’s Exact or Fisher-Freeman-Halton tests, depending on the expected value principle. The Pearson’s or Spearman’s correlation analysis was used to examining correlations, depending on the type of data distribution. Descriptive statistics were expressed as mean ± standard deviation, median, quartiles, and minimum-maximum values depending on the type of data distribution, while categorical variables were summarized as numbers and percentages. Statistical analyses were performed using the SPSS version 22 and the level of significance was set to *p* < 0.05.

## Results

The mean age was 12.00 ± 3.30 years in the patient group and 12.11 ± 3.16 years in the control group. The sex distribution was similar between the patient and control groups (Table 1). All patients had at least one mutation in the MEFV gene, while some patients had multiple mutations (Table 1). The mean duration of disease remission was 5 months, with a range of 3–6 months. Despite clinical remission, the patient group had statistically significantly higher amyloid, fibrinogen and C-reactive protein levels, and ESR and spot urine protein-to-creatinine ratio than the control group (*p* < 0.001) (Table 2). The corrected intraocular pressure was 14.96 ± 2.61 mmHg in the FMF group and 14.61 ± 2.15 mmHg in the control group, with no significant difference between the groups.

**Table 1:** Demographic and genetic characteristics of the patient and control groups.

	Patient (*n* = 51)	Control (*n* = 51)	*p*
Age (year)	12.00 ± 3.30	12.11 ± 3.16	0.867
Sex, *n* (%)			
Girl	31 (60.8)	31 (61.0)	0.937
Boy	20 (39.2)	20 (39.0)	
ISSF score (*n*)	≥6: 16		
	3–5: 15	–	–
	<3: 20		
M694V			
heterozygous	11 (21.6)	0 (0.0)	**0.001**
E148Q			
heterozygous	7 (13.7)	0 (0.0)	**0.014**
R202Q			
heterozygous	28 (54.9)	0 (0.0)	
homozygous	9 (17.6)	0 (0.0)	**<0.001**
A744S. E167D. F479L			
heterozygous	2 (3.9)	0 (0.0)	0.497
L110P			
heterozygous	1 (2.0)	0 (0.0)	0.999
L110P			
heterozygous	3 (5.9)	0 (0.0)	0.245
V726A			
heterozygous	3 (5.9)	0 (0.0)	0.245
P369S			
heterozygous	3 (5.9)	0 (0.0)	0.245
R408Q			
heterozygous	4 (7.8)	0 (0.0)	0.120
M680I			
heterozygous	2 (3.9)	0 (0.0)	
homozygous	1 (2.0)	0 (0.0)	0.497

Bold values indicate statistical significance *p* < 0.05.

**Table 2:** Laboratory parameters of the patient and control groups.

	Patient (*n* = 51)	Control (*n* = 51)	*p*
Amyloid (mg/dL)	2.37 (6.21) [0.02–177.00]	0.04 (0.04) [0.01–0.09]	**<0.001**
Fibrinogen (mg/dL)	286.55 ± 46.02	223.87 ± 26.58	**<0.001**
CRP (mg/dL)	0.09 (2.84) [0.01–16.95]	0.07 (0.03) [0.06–1.50]	**<0.001**
ESR (mm/h)	16 (21) [1–82]	12 (6) [3–20]	**<0.001**
Proteinuria	0.12 (0.10) [0.05–5.10]	0.09 (0.11) [0.01–0.21]	**<0.001**

*CRP* C-reactive protein, *ESR* erythrocyte sedimentation rate.

Bold values indicate statistical significance *p* < 0.05.

The SD-OCT measurements revealed that the temporal inferior RNFL was significantly thinner in the patient group than in the control group (*p* = 0.008). Moreover, although the central macular thickness and submacular choroidal thickness were lesser in the patient group than in the control group, but the difference was statistically insignificant. There was no statistically significant difference in AL between the groups. On the other hand, the thickness at nasal (ediN_500_, ediN_1000_, ediN_1500_), temporal (ediT_500_, ediT_1000_ and ediT_1500_) were also lesser in the patient group than in the control group; however, the difference was statistically insignificant. After grouping the patients according to ISSF scores, there was no significant difference in the results of SD-OCT measurements between the groups according to the disease score. The ocular findings of the patient and control groups are shown in Table 3.

**Table 3:** Ophthalmic findings of the patient and control groups.

	Patient (*n* = 51)	Control (*n* = 51)	*p*
AL (mm)	23.21 ± 0.81	23.00 ± 0.88	0.222
RNFL-T (μm)	70.82 ± 11.01	74.11 ± 10.08	0.132
RNFL-T_S_ (μm)	142.45 ± 21.11	145.13 ± 17.57	0.504
N_S_ (μm)	117.33 ± 24.25	112.60 ± 19.36	0.298
N (μm)	79.80 ± 11.35	77.07 ± 10.78	0.230
N_i_ (μm)	119.24 ± 24.38	114.47 ± 19.74	0.299
T_i_ (μm)	142.16 ± 18.90	152.20 ± 17.12	**0.008**
G (μm)	102.80 ± 11.05	103.27 ± 8.89	0.823
CMK (μm)	213.53 ± 14.79	217.64 ± 13.76	0.163
ediM (μm)	336.39 ± 81.28	353.38 ± 84.43	0.318
ediN_500_ (μm)	308.61 ± 76.75	331.44 ± 79.93	0.157
ediN_1000_ (μm)	285.69 ± 73.55	301.87 ± 80.71	0.307
ediN_1500_ (μm)	259.67 ± 71.92	268.47 ± 80.70	0.573
ediT_500_ (μm)	336.53 ± 79.28	353.80 ± 86.10	0.309
ediT_1000_ (μm)	335.84 ± 78.83	341.56 ± 79.41	0.725
ediT_1500_ (μm)	326.02 ± 79.06	336.42 ± 79.91	0.524

*AL* axial length, *RNFL* Peripapiller retinal nerve fiber layer thickness (*G* global, *T* temporal, *T*_s_ temporal superior, *T*_i_ temporal inferior, *N* nazal, *N*_s_ nazal superior, *N*_i_ nazal inferior), *CMK* central macular thickness, *ediM* Enhanced depth imaging choroidal thickness under the macula.

Bold values indicate statistical significance *p* < 0.05.

While proteinuria and AL were positively correlated in the patient group, ediN_1500_ and fibrinogen level were negatively correlated (Table 4).

**Table 4:** Correlation between laboratory and spectral-domain optical coherence tomography analysis results of the patients.

Patients (*n* = 51)							
		Amyloid (mg/dL)	CRP (mg/dL)	Proteinuria		Fibrinogen (mg/dL)	ESR (mm/h)
AL (mm)	*r*_s_	−0.139	−0.010	0.282	*r*	−0.147	−0.150
	*p*	0.332	0.943	**0.045**	*p*	0.303	0.293
ACD (mm)	*r*_s_	−0.056	0.220	0.126	*r*	−0.101	−0.046
	*p*	0.699	0.120	0.378	*p*	0.483	0.747
RNFL-T (μm)	*r*_s_	0.355	0.050	−0.079	*r*	0.216	0.029
	*p*	**0.011**	0.728	0.580	*p*	0.127	0.841
RNFL-T_S_ (μm)	*r*_s_	0.199	0.332	−0.049	*r*	0.161	0.159
	*p*	0.162	**0.017**	0.730	*p*	0.259	0.266
N_S_ (μm)	*r*_s_	−0.040	0.087	0.140	*r*	−0.159	−0.075
	*p*	0.782	0.546	0.325	*p*	0.265	0.601
N (μm)	*r*_s_	0.108	−0.035	0.190	*r*	0.129	0.035
	*p*	0.449	0.806	0.183	*p*	0.367	0.808
N_i_ (μm)	*r*_s_	0.076	0.008	−0.140	*r*	−0.094	0.038
	*p*	0.594	0.956	0.327	*p*	0.512	0.790
T_i_ (μm)	*r*_s_	0.106	0.195	0.055	*r*	−0.036	0.073
	*p*	0.458	0.170	0.701	*p*	0.800	0.609
G (μm)	*r*_s_	0.214	0.207	0.023	*r*	0.041	0.061
	*p*	0.132	0.145	0.871	*p*	0.775	0.671
CMK (μm)	*r*_s_	−0.185	−0.151	0.275	*r*	−0.194	−0.139
	*p*	0.194	0.291	0.050	*p*	0.172	0.332
ediM (μm)	*r*_s_	0.092	−0.260	−0.149	*r*	−0.188	−0.064
	*p*	0.522	0.066	0.297	*p*	0.186	0.653
ediN_500_(μm)	*r*_s_	0.051	−0.146	−0.057	*r*	−0.232	0.044
	*p*	0.723	0.305	0.691	*p*	0.101	0.761
ediN_1000_(μm)	*r*_s_	0.012	−0.208	−0.110	*r*	−0.265	0.026
	*p*	0.933	0.144	0.442	*p*	0.060	0.859
ediN_1500_(μm)	*r*_s_	0.047	−0.159	−0.062	*r*	−0.285	−0.017
	*p*	0.746	0.265	0.668	*p*	**0.043**	0.903
ediT_500_(μm)	*r*_s_	−0.001	−0.155	−0.073	*r*	−0.212	−0.120
	*p*	0.993	0.277	0.610	*p*	0.135	0.401
ediT_1000_(μm)	*r*_s_	0.011	−0.158	−0.053	*r*	−0.208	−0.190
	*p*	0.938	0.267	0.714	*p*	0.142	0.183
ediT_1500_(μm)	*r*_s_	0.029	−0.141	−0.061	*r*	−0.239	−0.224
	*p*	0.841	0.324	0.669	*p*	0.091	0.114

*CRP* C-reactive protein, *ESR* erythrocyte sedimentation rate, *AL* axial length, *RNFL* Peripapiller retinal nerve fiber layer thikness (*G* global, *T* temporal, *T*_s_ temporal superior, *T*_i_ temporal inferior, *N* nazal, *N*_s_ nazal superior, *N*_i_ nazal inferior), *CMK* central macular thickness, *ediM* Enhanced depth imaging choroidal thickness under the macula.

Bold values indicate statistical significance *p* < 0.05.

## Discussion

This study aimed to investigate the ocular effects of chronic subclinical inflammation in patients with FMF and found that the peripapillary RNFL, as measured by SD-OCT, was thinner in the temporal inferior quadrant in the patient group. Furthermore, an inverse correlation was observed between serum amyloid levels and temporal RNFL thickness. Similarly, choroidal thickness was found to be lower in the patient group compared to the control group.

Even if there is no evident clinical involvement in chronic inflammatory diseases, changes occur in many organs and tissues. In FMF, the persistence of subclinical inflammation between attacks, in addition to the inflammation occurring during the attacks, contributes to the development of adverse effects on other organs and tissues. Such damage may occur through several described mechanisms and may also be associated with an increased risk of endothelial dysfunction and atherosclerosis resulting from chronic subclinical inflammation, even during remission.^1^

Elevated serum levels of TNF-α and other cytokines have been reported in certain chronic ocular disorders.^19^ A study by Tuzcu et al. demonstrated that temporal RNFL thickness is particularly vulnerable to inflammation and inflammatory cytokines. The researchers found a negative correlation between disease activity scores and temporal quadrant RNFL thickness in patients with ankylosing spondylitis. Their findings suggested that RNFL thickness was reduced due to the inflammatory effects of cytokines—particularly TNF-α—on the eye in ankylosing spondylitis, a chronic inflammatory disorder.^20^ In FMF, inflammation is known to persist not only during acute attacks but also throughout remission. In support of this, elevated serum levels of IL-1β and TNF-α were observed during remission compared to those in the healthy population.^21^ The decreased RNFL thickness observed in the present study suggests the presence of persistent inflammation, even during remission. The retina is one of the target organs due to its complex neurovascular structure. Photoreceptors located on the retinal pigment epithelium constitute the outermost layer, while the RNFL comprises the innermost layer.^22^ This layer is among the ocular structures most vulnerable to impaired supply resulting from inflammation. Therefore, microvascular damage induced by inflammation may result in the atrophy of these cells, leading to a reduction in RNFL thickness. The present study also evaluated RNFL measurements in relation to disease severity and found no significant difference in RNFL thickness among patients with mild, moderate, or severe scores. The absence of a statistically significant correlation between the change in RNFL thickness and the disease severity score suggests that the effect of subclinical inflammation may be more predictive of chronic damage than changes associated with acute attacks.

Previous studies investigating changes in choroidal thickness in FMF patients have reported inconsistent findings. A study by Alim et al. found no significant difference in choroidal thickness between FMF patients in remission and healthy controls.^23^ Gündoğan et al., on the other hand, demonstrated increased choroidal thickness in FMF patients during an attack compared to healthy controls. The authors attributed this observation to choroidal edema resulting from increased inflammation. They concluded that the systemic inflammatory and vasculopathic nature of FMF may lead to increased vascular permeability, exudation, and dilation of choroidal vessels, thereby contributing to increased choroidal thickness during acute attacks. However, the study was unable to ascertain whether the damage was permanent, as choroidal thickness was not evaluated during the remission period in the same patient group.^24^ Another study by Biçer et al. found that the choroidal thickness in the nasal quadrant was significantly lower in adult FMF patients than in the control group.^25^ In the present study, the choroidal thickness was lower in the patient group than in the control group. The thinner choroid observed in the study by Biçer et al. might be attributable to the adult study population and their prolonged exposure to chronic subclinical inflammation. The aforementioned study suggested that choroidal thinning in adults may be associated with atrophy. Chronic inflammation may impair the vascular integrity and function of the choroid. Ultimately, age-related atrophy may also impair the vascular integrity and function of the retinal layers. However, the researchers suggested that, since the patient and control groups were matched for age and sex, age-related influences were ruled out, and the observed changes were attributed to the disease. The choroid, with its rich vascular structure, is likely to reflect microvascular damage and tissue loss through its thinning. In addition, arteriosclerosis, a sign of chronic inflammation, may contribute to choroidal thinning as the disease duration increases. The absence of choroidal thinning in the present study may be attributed to the shorter disease duration in comparison to adults. However, choroidal thickness is also expected to decrease in pediatric patients with longer disease duration, irrespective of attacks. The absence of a difference in disease scores between the groups may also be attributed to the relatively short duration of the disease. With prolonged disease duration, it is believed that ocular findings may accompany systemic changes in patients with high disease activity scores.

All patients included in the study were receiving regular colchicine treatment. Colchicine, while effectively controlling FMF symptoms, has been associated with ocular toxicity, including retinal changes and corneal deposits. These effects should be considered when interpreting ophthalmic findings. Experimental studies suggest that colchicine can interfere with or delay the repair of nerve structures after injury. Dybowski et al. reported that administering colchicine intraocularly inhibits the regeneration of retinal nerve fibers. They proposed that the formation of new microtubules is essential for nerve growth and that colchicine exerts its effect by selectively binding to the β-tubulin unit of the α/β-tubulin heterodimer, thereby preventing the incorporation of additional heterodimers into the microtubule structure. They noted that this effect occurs shortly after intraocular colchicine injection, and that any unbound colchicine is subsequently cleared and inactivated via systemic circulation. Considering that patients in the study group received colchicine at therapeutic doses and exhibited no signs of toxicity, we believe that systemic clearance of unbound colchicine likely limits its impact on nervous tissue.^26^

The kidney and eye are quite similar in developmental, structural and pathogenic pathways. Given the structural and functional similarities between renal podocytes and retinal pericytes, the pathological processes affecting these organs may also overlap.^27^ Renal microvascular alterations play a key role in the pathophysiology of kidney injury and are among the primary contributors to proteinuria in kidney disease.^28^ A study by Balmforth et al. demonstrated an association between glomerular inflammatory damage and choroidal thinning.^22^ The authors interpreted choroidal thinning as a marker of systemic inflammation with renal involvement, reporting a strong correlation between increased proteinuria levels and reduced choroidal thickness. These findings indicate that retinal and choroidal changes observed in CKD may reflect widespread systemic microvascular damage. The present study revealed a significantly higher incidence of proteinuria in the patient group compared to controls, despite values remaining within normal limits. This suggests that in FMF, increased protein excretion—and consequently, early renal involvement—may occur even in the absence of amyloidosis. However, no significant association was found between proteinuria and RNFL thickness. A previous study reported that retinal thinning in patients with chronic inflammation predominantly affects the outer retinal layers.^22^ This finding also implies choroidal involvement, as the outer one-third of the retina is nourished by the choroid, while the inner two-thirds are supplied by the central retinal artery. Although choroidal damage may impact retinal structure, the absence of a significant association between proteinuria and RNFL thickness suggests that alternative, non-vascular mechanisms may be involved. Inflammation leads to direct tissue damage in the eye prior to vascular involvement, meaning that vascular damage, which contributes to increased proteinuria, may not influence RT. Consistent with this, elevated fibrinogen levels—an important inflammatory marker—and the negative correlation between fibrinogen and choroidal thickness further suggest that inflammation plays a key role in inducing significant choroidal alterations.

On the other hand, it has been shown that AL levels increase in parallel with protein excretion in FMF. The simultaneous involvement of both the kidney and the eye suggests that inflammation may affect these organs via shared pathological pathways. The NLRP3 inflammasome (NACHT, LRR, and PYD domain-containing protein 3) is a macromolecular cytoplasmic complex that regulates the innate immune system’s early inflammatory response through the production of IL-1β and IL-18. Activation of NLRP3 is a key factor in the kidney injury process, including the development of proteinuria.^29^ Additionally, NLRP3 has been implicated in the inflammatory processes of other organs. Studies have shown that inhibiting NLRP3 in certain inflammatory conditions, including retinal injury and involvement of other ocular layers, can restore several ocular findings, particularly retinal neovascularization, by blocking the IL-1β/IL-18 activation pathway.^30^ An experimental study demonstrated significantly elevated IL-1β levels in MEFV gene-positive mice, even in the absence of active disease.^31^ Similarly, elevated IL-18 levels have been reported in both the acute attack and remission phases of FMF patients.^32^

Given these findings, it is plausible that shared inflammatory mechanisms—particularly those mediated by NLRP3 activation—may contribute to both renal and ocular alterations in FMF. Although we observed a positive correlation between proteinuria and AL, no significant associations were found between proteinuria and RNFL or choroidal thickness. This correlation may reflect shared microvascular injury mechanisms affecting both renal and ocular tissues in the early stages of FMF.

This study has several limitations. The patients were evaluated only during remission, which limits our understanding of inflammation-related ocular changes during acute attacks. A subgroup analysis based on remission duration was not feasible due to the small sample size, though most patients were in prolonged remission under regular colchicine treatment. While colchicine itself may affect ocular tissues, including untreated patients for comparison was not ethically possible. Future studies with larger cohorts are needed to investigate the cumulative effects of subclinical inflammation and to explore colchicine-related ocular changes more effectively. Although our study did not focus on the effects of specific MEFV mutations, future research into genotype–phenotype correlations may provide insights into FMF-related ocular involvement. Additionally, analyzing inflammatory markers and inflammasome activity during both remission and attack phases, as well as accounting for confounding factors like diet, medications, and environmental influences, could enhance our understanding of disease mechanisms. Long-term, multi-center studies following patients into adulthood are also warranted to better clarify FMF pathogenesis and potential therapeutic targets. Although this was a prospective study, the relatively short follow-up period may limit the ability to observe the long-term cumulative effects of subclinical inflammation. Therefore, future longitudinal studies with extended follow-up are warranted to better understand the progression of ocular changes in FMF.

The results of this study indicate that chronic inflammation can lead to thinning of the RNFL and choroid, even in childhood, when the duration of disease exposure is relatively short. Moreover, these findings suggest that the ocular manifestations of this chronic disease may be more diverse and long-lasting than previously anticipated. The observation that ocular changes in the patient group were present during the remission period and were not influenced by disease activity may be attributed to elevated levels of certain inflammatory markers, which persist even in remission. However, it is important to note that other inflammatory factors, which increase during active disease and return to normal during remission, also play an important role in the pathogenesis of FMF. These inflammatory markers, although elevated during active periods, may not have contributed to permanent damage due to the relatively short disease duration in the study participants. However, in a longer disease course, however, these inflammation-related factors could potentially lead to irreversible damage.

## Data Availability

All data generated or analyzed during this study are included in this published article [and its supplementary information files].
